# Perineural invasion as a neuro−immune niche in head and neck cancer: mechanisms of immune evasion and therapeutic implications

**DOI:** 10.3389/fimmu.2025.1719675

**Published:** 2025-12-09

**Authors:** Simin Li, Hui Xiao

**Affiliations:** Stomatological Hospital, School of Stomatology, Southern Medical University, Guangzhou, Guangdong, China

**Keywords:** perineural invasion, perineural tumour spread, head and neck cancer, adenoid cystic carcinoma, neuro− immune niche, nerve–tumour crosstalk

## Abstract

Perineural invasion (PNI) is a distinct route of spread in head and neck cancer and portends recurrence despite optimized surgery–radiotherapy backbones. Emerging cancer−neuroscience and immuno−oncology insights reframe PNI as a neuro−immune niche, in which neurotrophic signaling (GDNF–RET, NGF–TrkA), Schwann−cell plasticity, and neuromodulators (adenosine via CD39/CD73–A2A; nociceptor−derived CGRP) calibrate tumor behavior and immune tone along cranial nerves. Evidence spanning clinical and experimental models shows matrix−dominated, immune−excluded interfaces; adjuvant radiotherapy and nerve−pathway−directed target design mitigate local risk, while agents (A2A antagonists, TrkA inhibitors, CCR5 blockade) may counter perineural programs. Notably, deployment as immunity enablers remains uncertain, with unresolved issues in PNI quantification, biomarker selection, pharmacodynamic monitoring, and combinations with checkpoint or cellular therapy. In this review, building on cancer−neuroscience frameworks, we synthesize mechanistic drivers of the perineural niche, appraise efficacy− and safety−oriented pathology/imaging readouts and drugging opportunities spanning neural and immunometabolic circuits, and outline sequencing with locoregional measures to restore durable immunity. In addition, we discuss how nociceptor signaling and adenosinergic signaling intersect with Schwann−cell programs to regulate the balance between perineural immunosuppression and anti−tumor immunity. The purpose of this article is to define PNI as a neuro−immune niche in head and neck cancer and delineate biomarker−guided therapeutic strategies for clinical testing.

## 1 Introduction to perineural invasion in head and neck cancer

Perineural invasion (PNI) denotes the histopathological presence of malignant cells within, around, or along the sheaths of peripheral nerves and constitutes a distinct route of tumour propagation in head and neck cancer that is mechanistically and clinically separable from lymphatic or haematogenous dissemination (1–3). In routine practice, PNI is defined on microscopy, whereas perineural tumour spread (PNTS) refers to macroscopic extension along named nerves that is often detected on imaging; although the two entities are pathologically distinguishable, they form a biological continuum that underpins cranial neuropathies and skull−base involvement in advanced disease (4–6). In the head and neck region, PNTS most frequently involves branches of the trigeminal (V2–V3) and facial nerves, with retrograde extension to the skull base and cavernous sinus (7–9); We propose a practical PNI-burden framework extent (focal/multifocal/nerve calibre), depth (epineurial/perineurial/endoneurial), and trajectory (named-nerve concordance or imaging-defined PNTS) to guide reporting and treatment.

Across histologies, PNI is enriched in oral cavity HNSCC (immune-inflamed/adenosine-biased niches), adenoid cystic carcinoma (neurotrophin/sympathetic-coupled tracking), and cutaneous SCC (matrix/EMT-dominated, often nerve-field–centric) each linked to recurrence, nodal failure, and reduced survival (10–12). Historical and contemporary series, together with multi−institutional analyses, identify PNI as an independent high−risk pathological feature, and support escalation of locoregional therapy or margin−directed re−resection when feasible (13, 14). For early−stage oral cavity disease, adjuvant radiotherapy can attenuate the otherwise negative prognostic impact of PNI, underscoring the importance of accurate reporting and risk−adapted treatment selection (15–17). In cutaneous head and neck carcinomas, even a relatively low incidence of PNI (approximately 2–6%) carries disproportionate clinical significance, especially for mid−face tumours with proximity to cranial nerves (18, 19). PNI has also been linked to severe functional pain phenotypes in head and neck squamous cell carcinoma (20, 21), reflecting direct neural engagement by tumour and highlighting the need for integrated oncologic and symptom−directed management.

Mechanistically, PNI emerges from bidirectional trophic and chemotactic interactions between cancer cells, Schwann cells and neurons, superimposed upon permissive stromal and metabolic conditions. RET-dependent tumor-intrinsic chemotaxis toward GDNF drives directional migration, whereas nerve-driven neuritogenesis (axon outgrowth toward tumor) supplies contact guidance; co-culture microchannels quantify chemotaxis by cancer-cell flux to nerve-conditioned gradients, and ex vivo nerve-slice or microfluidic axon-outgrowth assays attribute path length and speed to neuritogenesis (22–24). Tumour−associated Schwann cells undergo phenotypic reprogramming, secrete guidance cues, remodel extracellular matrix and provide substrate for cancer cell locomotion along nerve tracts, thereby amplifying neural niche formation (25–27). Experimental platforms that recapitulate nerve–tumour interfaces (co−culture systems, microfluidic chambers and ex vivo nerve models) have refined causal links between axon−guidance pathways and directed invasion, laying a foundation for rational target discovery in head and neck cancer.

Converging evidence indicates that PNI does not operate in isolation from immunity; rather, nerves, glia and malignant cells co−create a specialised neuro−immune niche that conditions antigen presentation, leukocyte trafficking and effector function within perineural compartments (28–30). Neurotransmitters, neurotrophins and axon−guidance molecules modulate myeloid polarisation and T−cell activity, while inflammatory mediators reciprocally influence neuritogenesis and neural plasticity, collectively favouring tumour persistence along neural routes (31, 32). Recognising PNI as a neuro−immune locus is consistent with contemporary frameworks in cancer neuroscience and immuno−oncology and motivates integrative therapeutic strategies that combine precise locoregional control with modulation of neural and immune signalling (33, 34). This conceptual orientation aligns with emerging neuro−immune paradigms in solid tumours and provides a stylistic and methodological bridge to prior work that viewed cancer progression through a neuro−immune lens. The purpose of this mini−review is to synthesise current knowledge on perineural invasion in head and neck cancer as a neuro−immune niche, delineate its roles in immune evasion, and appraise therapeutic strategies that target this crosstalk to improve clinical outcomes.

## 2 The perineural tumour–nerve immune niche in head and neck cancer

Within head and neck cancers, perineural invasion establishes a spatially discrete micro-environment in which malignant epithelium, sensory and autonomic (adrenergic/cholinergic/peptidergic) fibers, Schwann cells, endoneurial/perineurial stroma and immune infiltrates interact; each fiber class differentially shapes local immunity (e.g., nociceptor-CGRP, sympathetic catecholamines) and fibroblasts under anatomical constraint (35–37). The multilamellar nerve sheath and endoneurial compartments provide low−resistance trajectories and diffusion−limited spaces that modify antigen sampling and cytokine gradients, rendering the perineural milieu immunologically and metabolically distinct from adjacent tumour–stroma interfaces (38–40). In this context, perineural invasion (a microscopic histopathological finding) and perineural tumour spread (macroscopic extension along named cranial nerves) are pathobiologically linked phenomena, most often involving branches of the trigeminal and facial nerves, and together constitute the physical substrate for a specialised tumour–nerve immune niche in the head and neck.

The niche is actively assembled by bidirectional trophic and guidance programmes rather than serving as a passive conduit. Nerve−derived cues, notably glial cell line−derived neurotrophic factor (GDNF), chemoattract head and neck squamous carcinoma cells via RET−dependent signalling and matrix−remodelling programmes, while tumour−derived neurotrophins such as nerve growth factor (NGF) engage TrkA to reinforce migratory and epithelial–mesenchymal transition states (41–43). Concurrently, Schwann cells adopt repair−like phenotypes, express neural cell adhesion molecules, remodel laminin−rich matrices and physically chaperone cancer cells along neurites in contact−dependent fashion—behaviours shown to accelerate neural tracking and dispersion of cancer cells (44, 45). Axon−guidance systems (semaphorins and related ligands) are variably co−opted in head and neck malignancies and interface with both motility and local immune regulation, further shaping the perineural ecology (46, 47). The principal structural, cellular and soluble elements that organise this niche in head and neck cancer are shown for **Table 1**.

**Table 1:** Constituents, mediators and functions of the perineural tumour–nerve immune niche in head and neck cancer.

Element	Principal constituents	Representative mediators/receptors	Functional consequences within the perineural niche
Nerve fibres	Sensory and autonomic axons; endoneurial microvessels	Neurotransmitters (e.g., norepinephrine, acetylcholine), neuropeptides (e.g., substance P, CGRP), neurotrophins (GDNF, NGF)	Chemotaxis and growth support for tumour cells; modulation of local vascular tone and leukocyte trafficking; peri-axonal metabolic exchange
Schwann cells	Myelinating and non-myelinating (repair-like) glia	NCAM1, laminins, MMP2/9; trophic ligands (e.g., GDNF)	Guidance and physical scaffolding for tumour locomotion; extracellular-matrix remodelling; conditioning of immune cells through cytokine release
Nerve sheath stroma	Perineurium, endoneurium, basal lamina	Tight-junction proteins; collagen IV, laminin, fibronectin	Diffusion barriers and anisotropic ECM tracks that favour directed migration and limit immune cell penetration
Malignant epithelium	HNSCC/ACC cells at nerve–tumour interface	RET, TrkA, p75^NTR^; integrins (e.g., α6β4); proteases	Neurotrophin and guidance cue sensing; perineural entry through proteolysis and adhesion dynamics
Immune compartment	Macrophages, dendritic cells, T cells (effector and regulatory), mast cells, neutrophils	Adenosine (CD39/CD73–A2A axis), IL-6/TNF family cytokines, chemokines	Myeloid-skewed infiltration; effector-T-cell restraint and impaired antigen presentation; reciprocal promotion of neuritogenesis
Microvasculature/lymphatics	Endoneurial capillaries; perivascular cells	VEGF/ANG signalling; adhesion molecules	Nutrient/oxygen supply in confined spaces; privileged trafficking corridors; potential egress routes for immune cells
Metabolic context	Hypoxia, acidosis, high extracellular ATP/adenosine	HIF-dependent programmes; ectonucleotidases	Reinforcement of immunosuppression and maintenance of low-shear, permissive conduits for spread

The immune dimension of the perineural niche is characterised by myeloid-dominant infiltrates, with focal B-cell/TLS aggregates contiguous with neural tracts (biomarkers: CXCL13, CD20/CD21L; exploratory anti-myelin proteins), and metabolically enforced immunoregulation. In head and neck squamous cell carcinoma, adenosinergic signalling (via CD39/CD73 and the A2A receptor) is germane to perineural compartments; in human specimens this can be assayed by multiplex IHC (CD39, CD73, A2A with CD8/FOXP3) (47), RNA-ISH for NT5E/ENTPD1, spatial metabolomics for adenosine/ATP ratios, and ex vivo A2A-pharmacodynamic readouts (pCREB in T cells) (48). Composition of the broader oral cavity tumour micro−environment—particularly reduced helper−T−cell density—also correlates with recurrence risk, consonant with an immune milieu that is permissive to local progression when effector support is scarce (49, 50). Gene−expression profiling of head and neck cutaneous squamous cell carcinoma with extensive perineural invasion demonstrates enrichment of epithelial–mesenchymal transition, adhesion and extracellular−matrix programmes, consistent with an immune−excluded, matrix−dominated state at the perineural interface (51, 52). Together with foundational observations that innervation modulates tumour immunity across solid cancers, these studies support the view that the perineural track functions as an immune−conditioned ecological niche rather than a purely anatomic pathway.

The niche’s anatomical specialisation carries clinical implications. The alignment of tumour cells with named cranial nerve pathways, the propensity for retrograde extension towards the skull base, and the imaging correlates of perineural tumour spread reflect an integrated biological continuum that merges directed neuritropism with local immune conditioning with dynamic blood–nerve barrier remodeling in tumor-adjacent nerves conditioning immune-cell entry and drug access features that must be accounted for in pathologic reporting and regional therapy design.

## 3 Immune−evasion programmes orchestrated by perineural invasion in head and neck cancer

Perineural invasion establishes an immune−evasive compartment by combining microanatomical constraint with stromal reprogramming. The multilamellar perineurium and collagen IV/laminin-rich, fiber-aligned ECM generate anisotropic pores and low chemokine permeability that impede dendritic-cell sampling and T-cell intravasation (26, 40, 42), while nerve-associated laminins engage integrins (α6β1/α7β1) and mechanosensors (YAP/TAZ, Piezo1/2) to bias tumor and immune-cell guidance along sheaths; the aligned sheath provides low-resistance routes for tumour movement (53, 54). Transcriptomic analyses of head and neck cutaneous squamous cell carcinoma with extensive perineural invasion demonstrate enrichment of extracellular−matrix, adhesion and epithelial–mesenchymal transition programmes, consistent with an immune−excluded, matrix−dominated state at the nerve–tumour interface.

**Figure 1:**
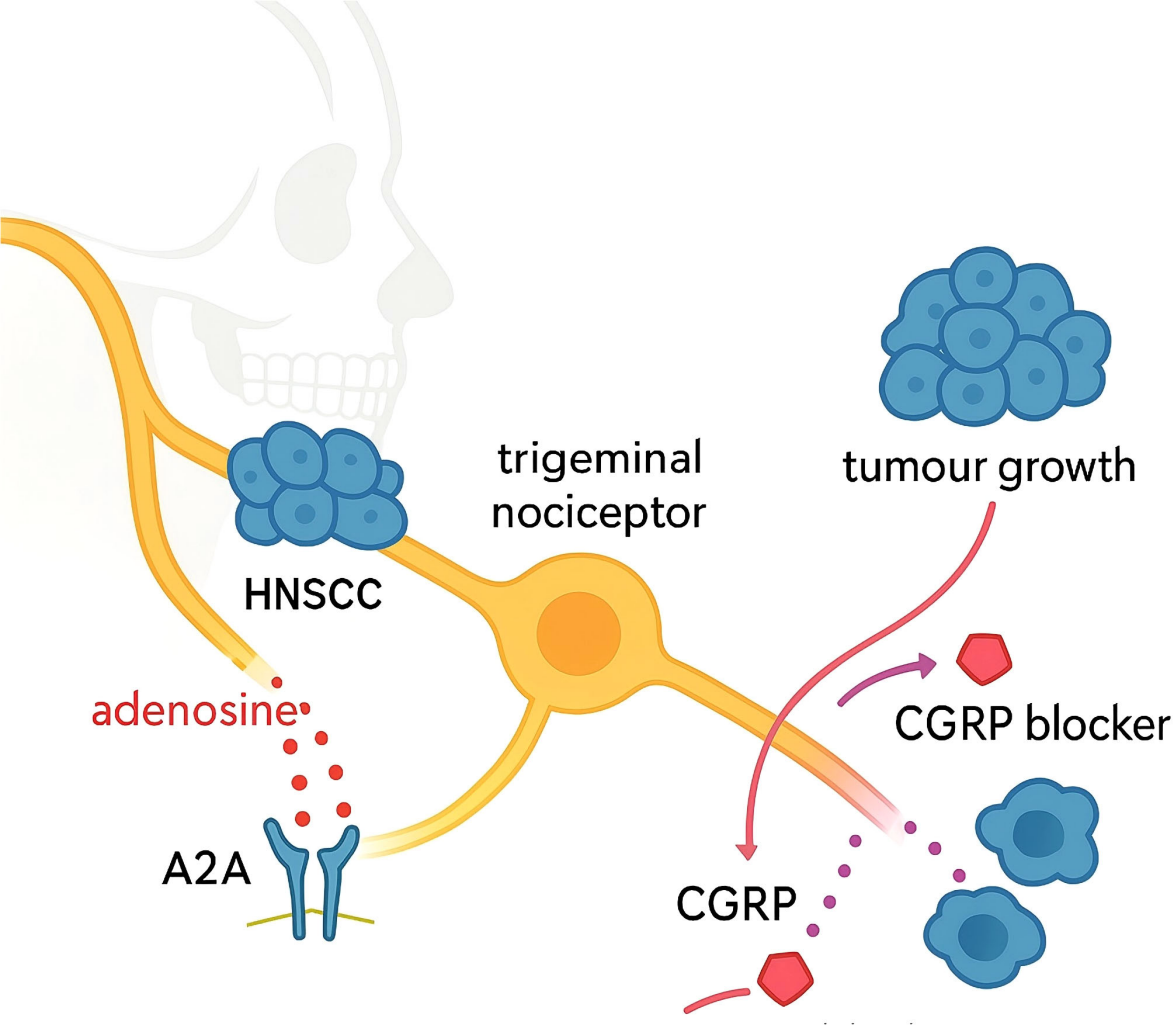
Adenosine A2A receptor CGRP signalling in trigeminal nociceptors promotes tumour growth and is targetable by CGRP blockade in head and neck squamous cell carcinoma with perineural invasion.

Neurochemical signalling within the perineural niche amplifies immunoregulation. As shown in **Figure 1**, in Head and Neck Cancer, adenosine accumulation driven by ectonucleotidases engages adenosine A2A receptors on trigeminal nociceptors, triggering calcitonin gene−related peptide (CGRP) release that accelerates tumour growth (55, 56); pharmacologic A2A antagonism or CGRP pathway blockade attenuates this effect *in vivo*, delineating a neuron−centred adenosinergic circuit that indirectly restrains antitumour immunity (57, 58). Complementary evidence from immunocompetent head and neck models shows that sensory-neuron-derived CGRP directly inhibits cytotoxic T-cell activity and reduces intratumoural CD8^+^ and Th1 CD4^+^ responses, and that microbial metabolites (SCFAs/indoles) with TLR2/4 engagement at mucosal–perineural interfaces may further modulate nociception and immune tone.

Glial participation further conditions local immunity. Although characterised most deeply in pancreatic ductal adenocarcinoma, single-cell and spatial profiling indicate that Schwann cells adjacent to tumour bundles acquire repair-like phenotypes that remodel matrix and instruct neighbouring stromal cells toward inflammatory fibroblast states via interleukin-1α and midkine signalling; they may also shuttle lactate/lipids to tumour cells (e.g., MCT1/4, FABP-mediated) (59), potentially co-varying with acidosis/adenosine-linked immunosuppression, and these changes co-localise with altered immune-cell composition compatible with perineural immune exclusion (60). In head and neck disease, clinicopathologic data reinforce the immunologic context: addition of tumour−microenvironment immune−cell classification to established staging identifies low helper−T−cell density as an independent predictor of recurrence in oral cavity squamous cell carcinoma (61–63), supporting the view that inadequate effector support promotes local progression along neural tracts.

These molecular and cellular programmes translate into clinically relevant resistance phenotypes. Perineural invasion is a high−risk pathological feature that portends adverse outcomes and necessitates escalated locoregional therapy, but it also coincides with immunosuppressive cues—most prominently adenosinergic signalling—that may blunt sensitivity to systemic immunotherapy. In recurrent or metastatic head and neck squamous cell carcinoma, higher CD73 expression on neoplastic cells correlates with early progression on immune−checkpoint blockade, consistent with an adenosine−conditioned microenvironment that diminishes effector function (64–66). Perineural invasion in head and neck cancer orchestrates a multi−layered immune−evasion programme—comprising structural exclusion, neuron−derived neuromodulators and glial−driven stromal remodelling—that should inform biomarker development and the design of combinations that concurrently target neural and immune axes.

## 4 Therapeutic levers targeting perineural neuro−immune crosstalk in head and neck cancer

Therapeutic strategies that interrupt the neuro−immune circuitry of perineural invasion should combine precise locoregional control with targeted modulation of neural, glial and immunometabolic pathways. For locoregional therapy, adjuvant radiotherapy mitigates the otherwise adverse prognostic effect of perineural invasion in early oral cavity squamous cell carcinoma and should be prioritized when pathological risk is present (67–69). Nerve−directed target delineation that explicitly contours involved cranial nerve pathways to the skull base is recommended to sterilize microscopic perineural tracks and reduce the risk of proximal failures (70–72). These principles are particularly relevant for mid−face and cutaneous primaries with radiographically occult spread, where predictable patterns of neural extension inform elective coverage of named nerves.

Pharmacologic interception of neurotrophin signalling represents a rational molecular lever. In head and neck squamous cell carcinoma, the NGF–TrkA axis…; anticipated on-target effects of NGF/TrkA inhibition include sensory paresthesias/hypoalgesia, mitigated by dose titration, intermittent scheduling, local-field delivery when feasible, and rescue with non-opioid analgesic algorithms (73, 74). In salivary adenoid cystic carcinoma, Schwann cell programs couple to tumour neurotrophin signalling; autophagic Schwann cells drive NGF–TrkA−mediated perineural invasion, indicating that glial−directed interventions (e.g., modulation of Schwann cell autophagy or adhesion programs such as NCAM1) may constrain neural tracking (75, 76). Chemokine circuits are also actionable; beyond CCL5–CCR5, CCL2/CCR2 and CX3CL1/CX3CR1 track perineural migration and macrophage/monocyte engagement, nominating a biomarker-first strategy (IHC/ISH or RNA panels for ligand–receptor pairs) to stratify trials to CCR2, CX3CR1, or CCR5 blockade where axes are activated (77, 78). In parallel, adrenergic stress signalling is implicated in head and neck tumour invasion; proof−of−concept studies in oral squamous cell carcinoma show that β−adrenergic receptor blockade is feasible and biologically active, providing a route to attenuate nerve−derived catecholaminergic inputs that condition the tumour–nerve interface.

Immunometabolic levers should be integrated where perineural niches exhibit adenosine−dominated suppression. Clinical correlative data in recurrent/metastatic head and neck squamous cell carcinoma indicate that higher CD73 expression on neoplastic cells predicts inferior response to PD−1−based immunotherapy, prioritising CD73 inhibition or A2A receptor blockade as combination partners to restore effector function in adenosine−rich microenvironments (79, 80). Within a treatment schema, these systemic agents can be sequenced with adjuvant or definitive radiotherapy to optimise antigen release and immune priming at the perineural interface, while nerve−axis inhibitors (e.g., TrkA−directed agents) and glial−modulating strategies are deployed to blunt chemotaxis and physical guidance along nerve sheaths (81, 82). Implementation should be biomarker−guided: pathological perineural invasion or imaging evidence of perineural spread, NGF/TrkA or CCR5 pathway activation, and elevated CD73 can anchor patient selection and stratification; field design that follows nerve topology ensures that locoregional measures are anatomically congruent with the biology of spread.

## 5 Conclusion and outlook

Perineural invasion (PNI) in head and neck cancer functions as a structured neuro−immune niche in which neuronal, glial and malignant programs converge to enable immune evasion and directed regional spread. Across histologies, PNI is a reproducible adverse factor for local control and survival, and its presence justifies treatment intensification in appropriately selected patients (83, 84). Mechanistic studies implicate neurotrophin and axon−guidance signaling (e.g., GDNF–RET, NGF–TrkA) and Schwann−cell reprogramming as drivers of neural tracking and extracellular−matrix remodeling at the nerve–tumor interface (85–87). The niche is further shaped by immunometabolic suppression through adenosine accumulation and CD39/CD73 activity, with downstream A2A signaling linked to diminished effector function (88, 89). Nociceptor−dependent release of calcitonin gene−related peptide (CGRP) can directly or indirectly impair antitumor T−cell responses and accelerate growth in head and neck models, situating sensory neurons as active regulators of perineural tumor ecology (90, 91). In clinical cohorts, higher CD73 expression correlates with early progression on PD−1–based therapy, consistent with an adenosine−conditioned micro−environment (92, 93). Collectively, these data support the interpretation of PNI as an anatomically constrained, immunosuppressed habitat that propagates disease along named cranial nerve pathways.

Clinical management should integrate rigorous locoregional control with targeted modulation of neural and immune axes. For early oral cavity disease with PNI, adjuvant radiotherapy mitigates the otherwise unfavorable prognosis and should be prioritized when feasible (94, 95). When perineural tumor spread is suspected or documented, nerve−pathway–directed target delineation to the skull base improves anatomic congruence between biology and field design and is recommended to reduce proximal failures (96, 97). Systemically, biomarker−guided combinations are warranted where perineural niches exhibit tractable vulnerabilities. In adenosine−dominant contexts, CD73 inhibition or A2A receptor blockade may restore effector function and augment checkpoint blockade (98–100). Where neuritropic programs are active, TrkA−directed strategies (or upstream NGF modulation) and, in salivary adenoid cystic carcinoma, interventions that disrupt Schwann−cell–coupled invasion merit evaluation (101, 102). In subsets with chemokine−dependent perineural migration, CCR5 antagonism is a plausible adjunct (103, 104). Implementation should be anchored by pathology and imaging evidence of PNI or perineural spread, pathway activation (e.g., NGF/TrkA, CCR5), and immunometabolic profiling (e.g., CD73), with prospective capture of pain phenotypes as clinically meaningful correlates of sensory−neuron engagement.

Methodological priorities include harmonized histopathologic reporting of PNI burden and location, and a conceptual MRI detection/segmentation pipeline (labels: named-nerve segments, perineural enhancement, foraminal fat effacement; quality targets: Dice ≥ 0.80 and case-level sensitivity; error modes: vascular mimics and post-treatment fibrosis), plus spatial immune profiling to quantify adenosine signaling, myeloid polarization and T-cell exclusion at the nerve–tumor interface. Trial designs should prospectively stratify by PNI status and perineural spread, mandate nerve−pathway–concordant radiation planning when indicated, and embed translational endpoints that measure neuromodulator dynamics (e.g., CGRP), ectonucleotidase expression and Schwann−cell phenotypes (105–108). Given the potential for immunosuppression to attenuate immune−checkpoint efficacy in CD73−high tumors, early−phase studies should evaluate sequencing and dosing that maximize antigen release and minimize neuromodulator−driven immune restraint, including rational radiotherapy–immunotherapy–neurotrophin/adenosine−axis combinations. A forward program should establish a multi−scale atlas of the perineural neuro−immune niche in head and neck cancer, link spatial pathway activation to clinical endpoints, and deploy biomarker-selected trials that couple nerve-pathway–informed radiation with NGF/TrkA, chemokine-axis, and adenosine agents, while prospectively mapping function-evoked, branch-specific pain (e.g., trigeminal V1–V3 maneuvers, alloknesis/thermal algometry) and integrating these phenotypes with spatial immune readouts (CD8/Treg, adenosine metrics). Integration of digital pathology for PNI quantification, MR−based nerve tractography for treatment planning, and longitudinal assessments of nociceptor activity and T−cell function will enable precise patient selection and adaptive therapy. Converging evidence indicates that aligning locoregional sterilization of nerve conduits with concomitant relief of immunometabolic and neuromodulatory suppression is a viable route to improve control and survival in PNI−positive disease.
